# Population Diversity Analysis Provide Insights into Provenance Identification of *Dendrobium catenatum*

**DOI:** 10.3390/genes13112093

**Published:** 2022-11-10

**Authors:** Xin-Yi Wu, Ting-Zhang Li, Fang Zheng, Jian-Bing Chen, Yue-Hong Yan, Jiu-Xiang Huang

**Affiliations:** 1 Key Laboratory of National Forestry and Grassland Administration for Orchid Conservation and Utilization, The Orchid Conservation and Research Center of Shenzhen and the National Orchid Conservation Center of China, Shenzhen Key Laboratory for Orchid Conservation and Utilization, Shenzhen 518114, China; 2 South China Limestone Plants Research Center, College of Forestry and Landscape Architecture, South China Agricultural University, Guangzhou 510642, China

**Keywords:** *Dendrobium catenatum*, genetic diversity, provenance identification, genuine regional drugs, RAD-seq, SNP

## Abstract

*Dendrobium catenatum* (*Dendrobium officinale*) is a valuable genuine herb. The source of this species is difficult to be identified by traditional methods including morphology, spectroscopy, and chromatography. We used the restriction site-associated DNA sequencing (RAD-seq) approach to perform the high-throughput sequencing of 24 *D. catenatum* provenances. In this study, 371.18 Gb clean data were obtained, and 655,057 high-quality SNPs were selected after their filtration. We used phylogenetic tree, genetic structure, and principal component analyses to examine the genetic diversities and genetic relationships of the 109 accessions. We found that *D. catenatum* could be divided into two groups, and each group was closely related to the distribution of the sampling sites. At the population level, the average nucleotide diversity (π) of the *D. catenatum* population mutation parameters was 0.1584 and the expected heterozygosity (*H*_E_) was 0.1575. The GXLPTP07 accessions showed the highest genetic diversity in terms of the private allele number, observed heterozygosity, and nucleotide diversity. The Mantel test showed a significant positive correlation between the genetic and geographic distances among the overall distribution. A genetic information database of *D. catenatum* was established, which confirmed that RAD-seq technology has the potential to be applied in the identification of medicinal *Dendrobium* of different origins.

## 1. Introduction

The genus *Dendrobium* Swartz (1799) is one of the largest genera in Orchidaceae [1], and it consists of approximately 1450 species [2]. *Dendrobium* is highly valued for its medicinal, ornamental, scientific and economics uses, but its medicinal value has attracted the most attention. There are nearly 80 species of *Dendrobium* in China. Many *Dendrobium* species have been extensively used in Traditional Chinese Medicine (TCM), including *D*. *catenatum* Lindl (*D*. *officinale* Kimura and Migo) and *D*. *huoshanense* C.Z. Tang and S.J. Cheng [3,4]. Many studies have shown that *Dendrobium* polysaccharide has antitumor effects [5], immune enhancement [6,7], antioxidation [8,9], anti-inflammatory [10,11], and hypoglycemic effects [12,13]. There is also research showing that *Dendrobium* can improve learning and memory [14]. The medicinal *Dendrobium* industry has developed rapidly in recent years, and some growers have carried out cross-breeding for medicinal *Dendrobium* in pursuit of increasing their yield and profits, which has caused confusion in the market regarding the *Dendrobium* varieties. There are great differences in the pharmaceutical components between *Dendrobium* species [15], which affect the efficacy or curative effect of them. A large number of medicinal *Dendrobium* products on the market have lost their active ingredients, and variable quality and effective identification methods are lacking. Traditional morphological identification, microscopic identification, and the physical and chemical identification methods can only be used for the interspecies identification of medicinal *Dendrobium* [16,17,18]. Obviously, it is difficult for conventional techniques to identify the provenance of medicinal *Dendrobium* as they are similar in their morphological and anatomical characteristics and even in their chemical components. One of the most important factors for the modernization of Chinese medicines is the quality of the traditional Chinese medicines, and the key support for it is the study of its genuineness [19]. A variety of genuine medicinal materials had been confirmed that the effective components of different producing areas are very different [20,21,22]. *D. catenatum* is a geo-authentic Chinese medicinal material, and so its identification of provenance is very important.

For identification purposes, researchers have established a full-sequence database that is based on the rDNA ITS region of the *Dendrobium* variety “Fengdou” [23]. The phylogenetic tree constructed from *matK* and *rbcL* data could distinguish five types of medicinal *Dendrobium* [24]. For the purpose of the swift and precise identification of thirteen wild and cultivated *Dendrobium* species belonging to two sections *Formosae* and *Chrysotoxae*, the researchers designed the rDNA ITS region sequence analysis [25]. In recent years, the molecular identification of *Dendrobium* plants has also been studied. Inter-simple sequence repeats (ISSR) molecular fingerprinting markers had been employed to authenticate eight populations of *D. officinale* using 10 primers [26]. Two genuine population had been authenticated based on the SNPs of the rDNA ITS region [27]. However, microsatellite studies have typically used a low number of markers (<25), which increases the danger of underestimating the genetic structure due to a lack of polymorphic markers [28]. When one is using a low number of genetic markers, larger sample sizes are required to accurately estimate the allele frequencies and diversity [29], which might be difficult to achieve, especially for *Dendrobium* as it is an endangered species that has been severely damaged. The SNPs produce more precise estimates of the population-level diversity, and they use a higher power to identify the groups in clusters than the methods of using microsatellites do [30]. To evaluate and quantify the potential marker-specific biases, researchers have compared the microsatellite variation with genome-wide SNPs. They concluded that a few thousand random SNPs are sufficient to accurately estimate the genome-wide diversity and to distinguish between the populations with different levels of genetic variation [31].

Currently, with the development of high-throughput sequencing technology, SNPs are increasingly used in species identification and population genetic research [32]. Compared with traditional molecular markers, the SNPs that are obtained by high-throughput sequencing can provide a large amount of accurate and reliable information for a genetic and evolutionary analysis [33]. Reduced representation genome sequencing has been widely used in the field of population research in areas such as population genetic analysis [34], marker development [35], genetic map construction and whole-genome association analysis [36,37]. These methods, which consist of restriction site-associated DNA sequencing (RAD-seq), have been successfully applied to the study of the population structure, genetic distance, and genetic diversity of many species [38,39,40,41]. Currently, high-quality *D. catenatum* genome data have been obtained and published [42]. Therefore, the RAD-seq of this species of medicinal *Dendrobium* can provide a large number of reliable SNPs to distinguish the provenances of them.

To protect the wild population of *D. catenatum* and meet the increasing market demand, the following protective measures are recommended: (a) in situ conservation; (b) building an ex situ conservation base; (c) establishing a provenance database to strictly control the use of wild populations while ensuring the reliability and detectability of their provenance. For the creation of management and conservation strategies, information on the genetic diversity and population structure are crucial. To better understand the genetic diversity, genetic organization, and divergence of the wild populations of *D. catenatum*, we produced and analyzed the SNPs for these populations using RAD-seq. Accurately identifying the provenances of *D. catenatum* based on RAD-seq will help growers to choose high-quality provenances for artificial cultivation and propagation. This research not only has important significance for the evolution, molecular breeding and biogeography of *Dendrobium*, but it also provides important enlightenment for the identification methods of other medicinal plants.

## 2. Materials and Methods

### 2.1. Sample Collection and DNA Extraction

**Figure 1:**
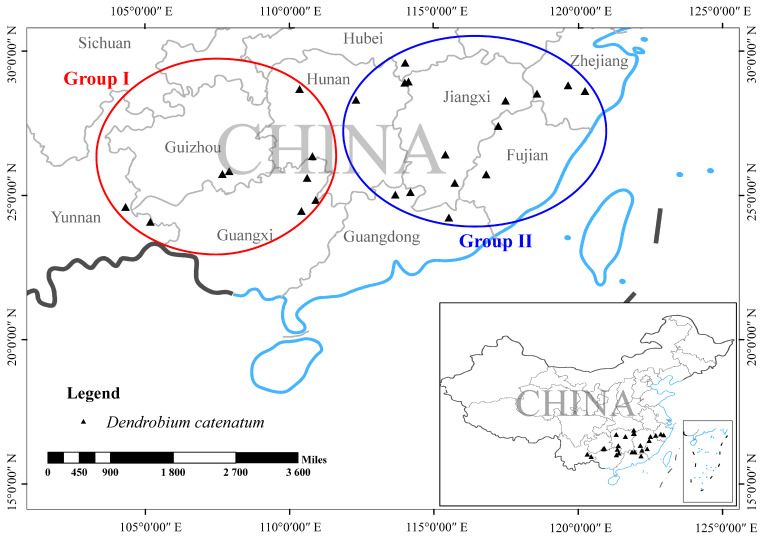
Sampling locations of *D. catenatum* species used in this study.

The plant materials that were sequenced in this study were collected from the National Orchid Conservation & Research Center of Shenzhen, and the specimens were deposited in the National Orchid Conservation Center herbarium (NOCC) (Table 1). All of the plant materials were collected with the permission of this institution, and all of the samples were identified by Prof. Zhong-Jian Liu. We complied with the IUCN Policy Statement on Research Involving Species at Risk of Extinction and the Convention on the Trade in Endangered Species of Wild Fauna. Detailed information about localities and samples are given in Table 1 and Figure 1. Specifically, we collected 109 samples were from 24 wild populations of *D. catenatum*, and 10 samples were from 2 wild populations of *D. huoshanense*. We selected 3–5 individuals from each population and sampled their leaves. The leaves were dried in sealed plastic bags that were filled with silica gel until the DNA extraction was performed.

The total genomic DNA was extracted using a Plant Genomic DNA kit (Tiangen, Beijing, China) according to the manufacturer’s protocol. For all of the samples, the DNA was quantified using a Qubit spectrophotometer (Invitrogen, Carlsbad, CA, USA).

### 2.2. RAD Library Development and Sequencing

The RAD sequencing libraries were generated using the VAHTS Universal DNA Library Prep Kit for Illumina (Vazyme Biotech Co., Ltd., Nanjing, China, ND604) following the manufacturer’s recommendations. In brief, the RAD-seq reduced representation libraries were prepared following the digestion procedure using the *Hae* III (New England Biolabs, Ipswich, MA, USA) enzyme, which was followed by a barcode ligation, a DNA purification and a selective DNA amplification and a size selection. Pair-end sequencing with a read length of 150 bp was performed to produce approximately 3 Gb of raw data for each sample using the Illumina HiSeq 4000 platform (Illumina, San Diego, CA, USA) at Novogene (Beijing, China).

### 2.3. SNP Calling

The raw Illumina paired-end reads were filtered using a personally designed program “filter-G 30.0-adapter 1-poly_A 1-Q 20,0.5-mean 25-insert $ insertSize-Q20 90” to discard the reads with adapter sequences, poly_A tails, a poor base quality (Q < 20), and those that were less than 25 bp in length. The cleaned reads were aligned to the *D*. *catenatum* genome (NCBI accession number NC_037361.1) using BWA (version 0.7.12) [43] default parameters, and they were sorted using Samtools (version 1.9), and following this, they were de-duplicated using the Picard tool [44]. In order to reduce the impact of mapping bias, we further excluded the sites with extraordinarily high or extremely low coverage. The RealignerTargetCreator and IndelRealigner modules from GATK (version 3.8) [45] were used to improve the local alignments around the indels. The resulting alignment files were subjected to genotyping using the GATK UnifiedGenotyper at each reference locus. Finally, hard filtering was applied to the raw variant set using GATK recommended parameters, “QD < 2.0 || MQ < 40.0 || FS > 60.0 || SOR > 3.0 || MQRankSum < −12.5 || ReadPosRankSum < −8.0”. We further filtered the dataset using VCFtools (version 0.1.13) [46] to ensure that we had high-quality SNPs, removing the SNPs with a missing rate > 20% and minor allele frequencies < 0.02.

### 2.4. Phylogenetic Tree Construction

The maximum likelihood (ML) analysis was performed using IQtree (version 2.0.3) [47] with 1000 bootstrap [48] replicates, and the settings were as described. The results were graphically visualized and edited in FigTree (version 1.4.2).

### 2.5. Population Structure Analyses

Genetic structure was investigated by a PCA. A PCA was conducted using GATK, and the program Admixture [49] was used to infer the genotype structure. A population number (K) ranging from 2 to 8 was assumed, and the CV scores were used to determine the best-fit K value.

### 2.6. Genetic Diversity and Differentiation

A variety of genetic diversity indices were calculated using the Stacks’ populations program, including the private allele number (A_P_), nucleotide diversity (π), heterozygosity (*H*_O_ and *H*_E_), and inbreeding coefficient (*F*_IS_) [50]. Sliding windows that were 10 kb in size were used to calculate Tajima’s D by VCFtools (version 0.1.13) [46]. To analyze the pairwise population differentiation between the wild germplasms, the *F*_ST_ values were also computed using the PopGenome package in R [51]. The pairwise geographic distances from longitude and latitude were identified using the R package geosphere. We used the Mantel test for the associations between the *F*_ST_ and geographic distance. PopLDdecay (version 3.31) was used to calculate the linkage disequilibrium between the SNP pairs within a 500 kb window [52]. The linkage disequilibrium decay was measured the distance at which the Pearson’s correlation efficient (r^2^) dropped to half of the maximum.

## 3. Results

### 3.1. Sequence Data Quality

For the 109 sequenced samples, 403.61 Gb of raw data with an average of 3.70 Gb per individual were generated, ranging from 1.74 to 5.29 Gb. After filtering the sequence data, a total of 371.18 Gb of clean data (1.61 Gb to 4.89 Gb for each individual, with an average of 3.37 Gb) was maintained, presenting an average effective rate of 91.97% (Supplementary Figure S1). In short, the sequencing data were of high quality and could be used for a subsequent analysis. Finally, 655,057 high-quality SNPs were selected after the filtration was performed. Nearly half of the SNPs in the population level exhibited base transitions, and the total transition-to-transversion (ts/tv) ratio was 1.36.

### 3.2. Genetic Diversity

When we were analyzing the variant positions for all of the polymorphic loci at the germplasm level, the observed heterozygosity (*H*_O_), expected heterozygosity (*H*_E_), nucleotide diversity (π) and Wright’s F-statistic (*F*_IS_) of the wild *D. catenatum* were 0.0992, 0.1575, 0.1584, and 0.3836, respectively (Table 1). The number of private alleles (A_P_) in the populations ranged from 1934 (FJLCTP02) to 9655 (GZDSTP30). The observed heterozygosity at the population level ranged from 0.0724 (GXXATP10) to 0.1516 (GZDSTP30); the expected heterozygosity for each population ranged from 0.0833 (FJLCTP02) to 0.1312 (GXLPTP07); the nucleotide diversity for each population ranged from 0.0961 (GDSGTP22) to 0.1484 (GXLPTP07); the inbreeding coefficient in each population ranged from −0.0434 (GZDSTP30) to 0.1135 (GXLPTP07).

Tajima’s D value was a locus-based indicator of the intraspecific polymorphism. Tajima’s D values were positive and statistically different from zero for all of the populations (Table 2). It is therefore possible to reject the null hypothesis of neutral evolution. In addition, many intermediate-frequency alleles were found in the populations, which may be a consequence of the bottleneck effects, the population structures, or the selection for balancing.

### 3.3. Phylogenetic Tree

**Figure 2:**
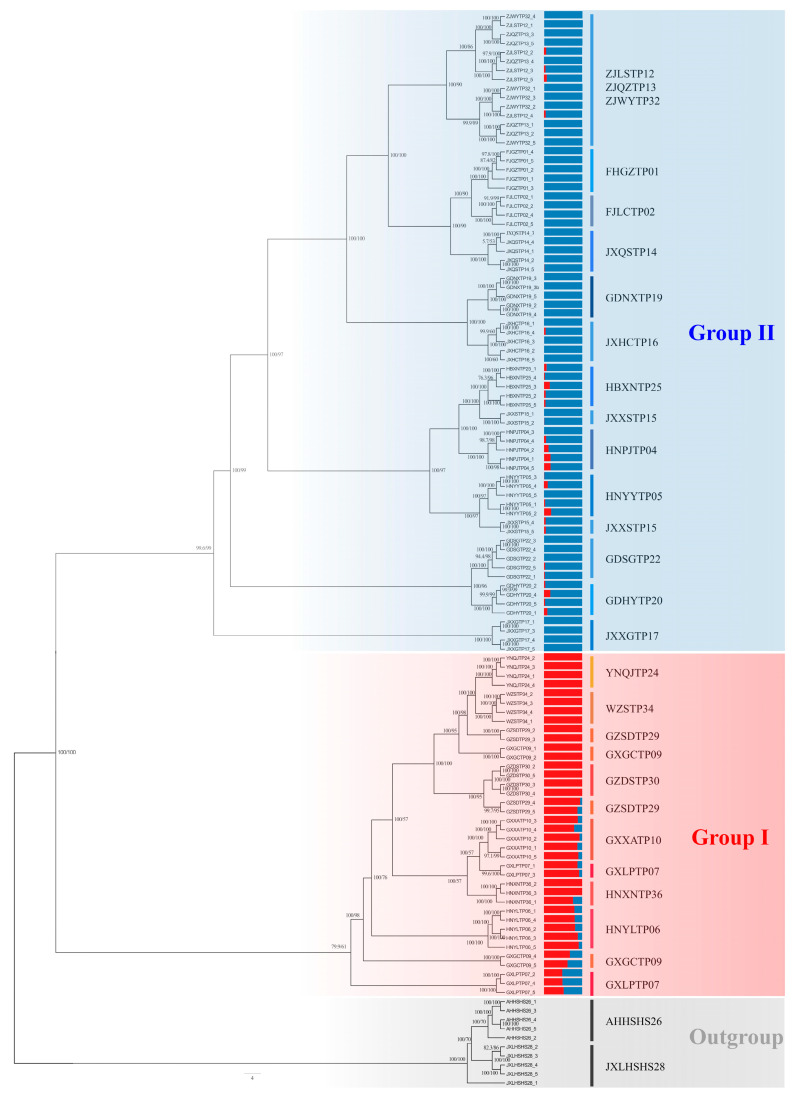
ML phylogenetic tree of the *D. catenatum* accessions and model-based clustering with K from 2. Numbers near the nodes are bootstrap percentages.

In the maximum likelihood (ML) phylogenetic tree that was derived from our finalized RAD-seq matrix, *D. huoshanense* was the outgroup (Figure 2). There were high bootstrap values on most of the branches of the phylogenetic tree, which indicated a strong reliability of the tree. All of the samples were separated into two clades based on the ML tree (Group I and Group II). Group I mainly contained *D. catenatum* from the Yungui Plateau and the western part of the Nanling Mountains, while Group II mainly contained populations from the Yandangshan Mountains, Wuyishan Mountains, and the eastern part of the Nanling Mountains.

In the Group I, the YNQJTP24 accessions were the sister group to the WZSTP34 accessions, which both had 100% bootstrap values. The samples from Guizhou Dushan (GZDSTP30), Guangxi Xing’an (GXXATP10) and Hunan Yiyang (HNYYTP06) are clustered into a branch. The samples from the provenances GZSDTP29, GXGCTP09, and GXLCTP07 were divided into two branches. In the Group II, for most of the provenances of *D. catenatum*, the individuals of the same provenance are clustered together with high support values, with the exception of the individuals of three populations from Zhejiang Wuyi (ZJWYTP32), Zhejiang Lishui (ZJLSTP12), and Zhejiang Quzhou (ZJQZTP13). The samples from Zhejiang were gathered into a large branch, and the sister branch was composed of FJGZTP01, FJLCTP02, and JXQSTP14. The populations from Hubei Xinning (HBXNTP25), Jiangxi Xiushui (JXXSTP15), Hunan Pingjiang (HNPJTP04), and Hunan Yiyang (HNYYTP05) are clustered into a large branch, and the other three provenances being independently clustered into a small branch, with the exception of JXXSTP15. The samples from Guangdong Shaoguan (GDXGTP22) and Guangdong Heyuan (GDHYTP20) are clustered into a branch. At the same time, the distribution of the *D. catenatum* samples in the phylogenetic tree are closely related to their geographical locations. The base of the phylogenetic tree is represented by the population that can be located in the west.

The geographical distribution of the *Dendrobium* samples from the west to the east is roughly consistent with their distribution from the base to the top of the phylogenetic tree.

### 3.4. Population Structure

**Figure 3:**
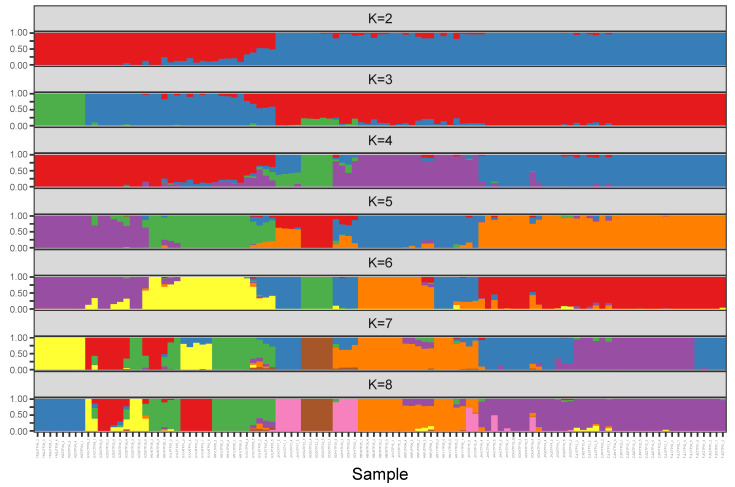
Genetic structure of cultivated 109 *D. catenatum* for K = 2–8 based on the Admixture software (K = 2 with cross validation error is 0.18580, while K = 3 with ross validation error is 0.19141).

**Figure 4:**
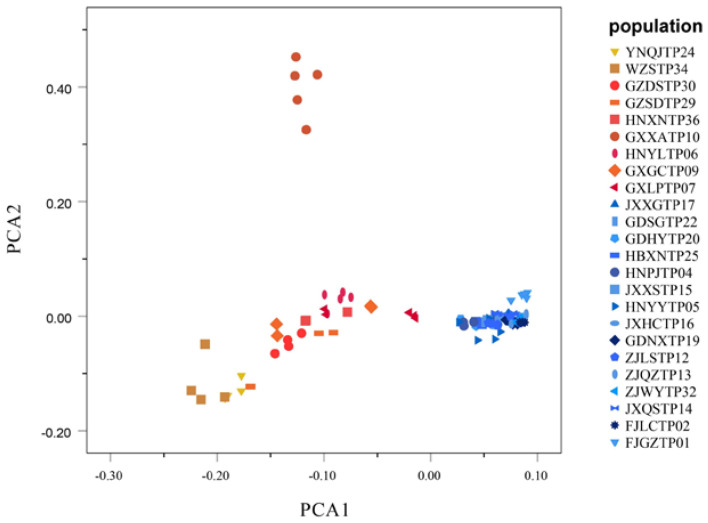
PCA of 109 samples in *D. catenatum*. Red shapes indicate Group I, while blue shapes indicate Group II.

Based on the ML tree (Figure 2), we calculated the population structure with K values that range from two to eight (Figure 3). In the clustering analysis using the Admixture software, K = 2 was the most likely genetic cluster number, because its cross-validation error (CV) was the lowest. However, the CV values were very close when K = 3 and K = 4. According to the principal component analysis (Figure 4), when the K value equaled two, 109 materials were not clearly assigned to the two groups, which was inconsistent with the results of the phylogenetic tree analysis. Due to the differences in the population structure and phylogenetic tree results, it was necessary to analyze the population structure under different K values.

When K = 2, the Group I and Group II accessions could be separated. When K = 3, the Yunnan populations in Group I were distinguished, which was inconsistent with K = 4. In conjunction with the phylogenetic tree and structure analysis, these results clearly classified *D. catenatum* into two groups.

### 3.5. Genetic Relationships

A principal component analysis (PCA) was performed with 109 individuals. According to the first component, the accessions were divided into two groups: Group I and Group II. Overall, GXXATP10 was clustered separately, and other *D. catenatum* accessions could be divided into two groups. Among them, the distribution of Group II was more concentrated (Figure 4).

**Figure 5:**
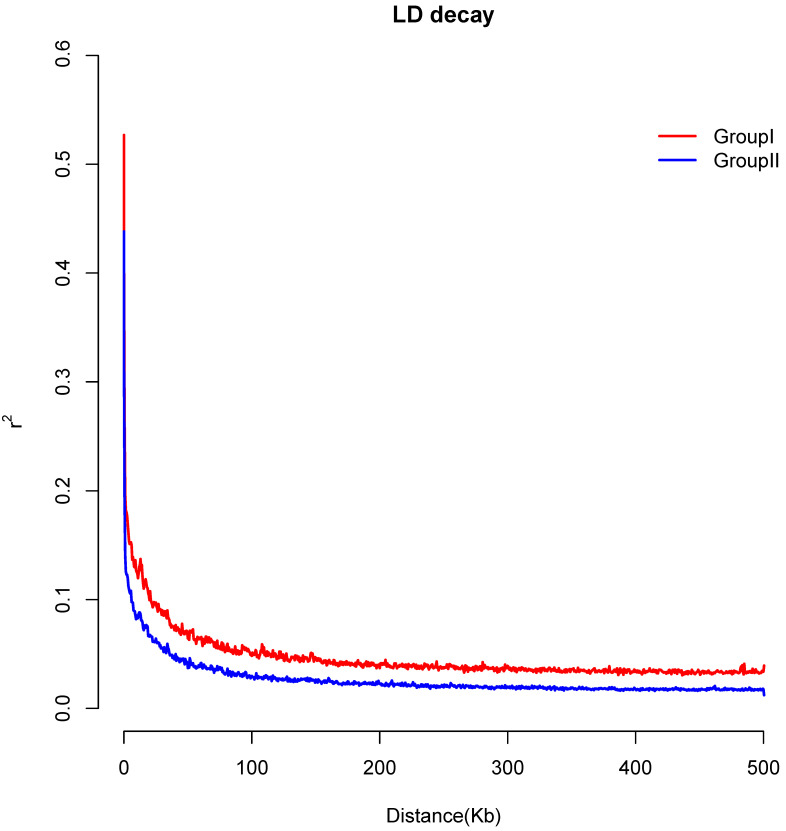
Linkage disequilibrium decay patterns of different *D. catenatum* group.

The linkage disequilibrium (LD) decay, which was measured by the physical distance, at which the pairwise correlation coefficient dropped to half of its maximum value, occurred at 48.2 kb in Group I (r^2^ = 0.277) and 13.6 kb in Group II (r^2^ = 0.242) (Figure 5), respectively. Group II had the higher linkage disequilibrium decay rate.

### 3.6. Genetic Differentiation

**Figure 6:**
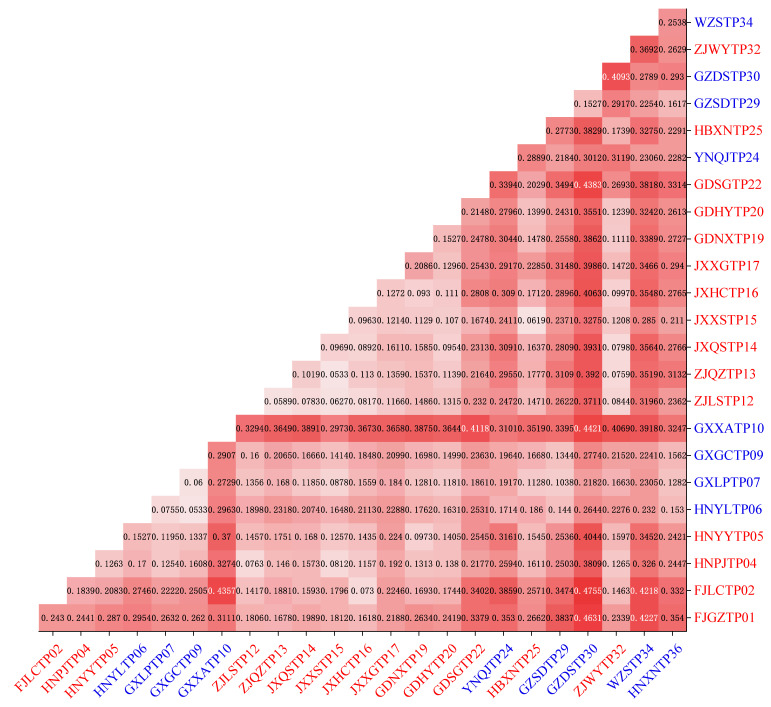
Genetic distance (*F*_ST_) for *D. catenatum*. The samples from Group I were in red font, and the samples from Group II were in blue font.

**Figure 7:**
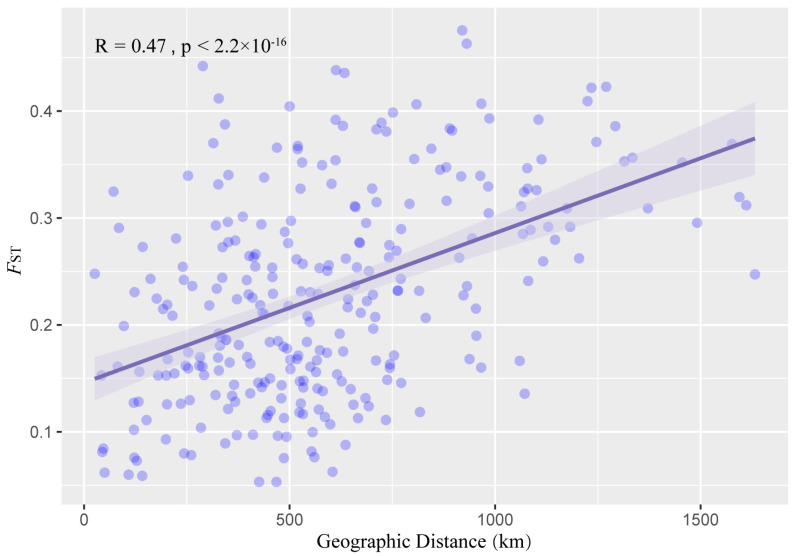
Relationships between the pairwise genetic distance (*F*_ST_) and geographic distance with blue bars and shading represent the 95% highest posterior density.

The pairwise *F*_ST_ values between the wild populations of *D. catenatum* varied from 0.0533 (HNYLTP06 with GXGCTP09) to 0.4755 (FJLCTP02 with GZDSTP30), with 110 of the 276 population pairs having *F*_ST_ values that are greater than 0.25 (Figure 6). It became clear that, GXXATP10 had the highest genetic differentiation from the other 24 populations which was consistent with the PCA results (Figure 4). GXLPTP07 was in the western part of the Nanling Mountains, and it obtained the lowest mean pairwise *F*_ST_ value, 0.1504; low values represent the genetic flow between the populations and show a greater genetic difference between the accessions within a population than it does between the populations. The pairwise genetic distance between the locations *F*_ST_ was highly correlated with the geographic distance (Figure 7); the level of significance was *p* < 0.05.

## 4. Discussion

Genomic data provide a novel perspective for studying the genetic diversity and method of identification of medicinal *Dendrobium* species. In the past, scholars have been authenticated eight wild populations of *D. officinale* by ISSR [26]. Because of the low number of microsatellite markers, marker ascertainment bias, and there being a high variance in the microsatellite-derived estimates, the genetic differentiation among the populations (*F*_ST_) estimates of microsatellites were significantly larger than those from the SNPs [31]. The simple PCR methods are simple and quite effective and cheap. However, their weakness is obvious at the same time. Firstly, these data are not reproducible enough, and it is difficult to share and reference them, and they can only be analyzed and used in the same experimental project. Secondly, these markers cannot be mapped to the genome for further analysis and applications. The data that are obtained by RAD not only have the advantages of being convenient and highly reproducible, but also, they can be accurately located in the genome position for more applications. For example, the candidate genes with known functions can be identified by annotating the outlier loci through the selection elimination analysis [53,54]. At the same time, the SNPs that are obtained by RAD-seq can provide more accurate and reliable information for a genetic and evolutionary analysis. Researchers utilized anadromous pike (*Esox lucius*) to assess the microsatellite and RAD-seq results of a study of population differentiation and genetic structure, and they discovered that the full RAD-seq dataset can provide the most accurate detection of the finer-scaled genetic structuring [55]. The nuclear microsatellites and the RAD-seq data for a threatened freshwater fish species were compared by other researchers. The results showed that RAD-seq more clearly and consistently identified the hierarchical phylogenetic structure [56].

In this research, a whole-genome restriction enzyme digestion of 109 accessions from 24 provenances was performed to obtain accurate variation information. Except for GXGCTP09, the clean data size of each sample in the other 23 populations was at least 2 times the *D. catenatum* genome size (1.11 Gb), and some individual populations reached 4 times this (Supplementary Figure S1). The average sequencing volume of each sample was 3.37 Gb. The amount of sequencing data was sufficient to meet the requirements of the subsequent analysis, thus ensuring the accuracy of population genetic analysis. In terms of the result of the RAD-seq, we detected 655,057 SNPs, which exceeded the number of genetic variations that were detected by amplified fragment length polymorphism (AFLP) and the random amplified polymorphic DNA (RAPD) markers [57,58]. These data not only can be used in this experimental analysis, but they are also convenient for researchers for other studies.

The number of transitions was predicted to be much larger than the number of transversions due to the biased mutational processes within the plant genomes. This nucleotide mutation pattern is also observed in other plants, such as peanuts [59], maize [60], *Amorphophallus paeoniifolius* [39], *Arabidopsis*, and apricots [61,62]. Consistent with this prediction, the ts/tv ratio of the *D. catenatum* populations was 1.36, indicating that there was a strong transition bias. This value was higher than the result (1.34) that was reported by Zhang et al. [42], and lower than that for *D. huoshanense* (1.47) [63].

Based on the ML tree, the principal component analysis, the genetic structure analysis, and the population differentiation analysis divided the 24 wild populations of *D. catenatum* into two groups. The two groups where the provenances of the wild *D. catenatum* are arranged from the west to the east are very consistent with the geographical distribution of the samples. In another study, Ding et al. used RAPD to study eight wild *D. catenatum* populations [58]. Among them, Shaowu and Shunchang in Fujian were clustered into a small branch, which was the sister branch of Jiangxi Nanfeng, and Tian’e in Guangxi and Guangnan in Yunnan were clustered into a small group. Our results in this study are consistent with the previous findings. In a systematic geographical study of *D. catenatum* and four related taxa, the regional evolution of *D. catenatum* was divided into six populations [64]. They were the South Yungui Plateau, the East Yungui Plateau, Nanling Mountain, Wuyishan Mountain, Dabieshan Mountain, and Yandang Mountain. In this study, Group I contained the Yungui Plateau and the western part of the Nanling Mountains, while Group II contained the Yandangshan Mountains, Wuyishan Mountains, and the eastern part of the Nanling Mountains. This is largely consistent with the division that had been made previously.

Genetic diversity forms during the evolution of a species, and it often plays a key role in the gradual evolution and long-term survival of the species in a changeable environment. The average nucleotide diversity (π) of the *D. catenatum* population mutation parameters was 0.1584 at the population level (Table 2). *D. catenatum* had a smaller mean nucleotide variation than other crops did such as *A. paeoniifolius* (π = 0.3592) [39]. At the population level, the expected heterozygosity (*H*_E_) was 0.1575, which was very close to the previous results (0.1477) [57]. Overall, the observed heterozygosity and the expected heterozygosity were relatively low.

Among all of the wild populations, GXLPTP07 had the most significant private allele number (AP) of 5221, and this indicated the presence of substantial genetic variation, which could guide genetic improvement in the future. The genetic diversity indices (*H*_E_, A_P_, and π) of the GXLPTP07 accessions were higher than those of the accessions in the other ecological groups (Table 2). We speculate that the GXLPTP07 was closer to the origin center of Group I in terms of genetic distance. In the principal component analysis, GXXATP10 was clustered separately. Combined with the analysis of genetic diversity, GXXATP10 had the highest Tajima’s D values, indicating that the population underwent a balanced selection or a sudden contraction. The results of the *H*_O_, *H*_E_, and inbreeding coefficient (*F*_IS_) show that *H*_O_ < *H*_E_, *F*_IS_ > 0, which may have resulted from the heterozygote loss and inbreeding, and the selection pressure is high (Figure 3, Table 2). Based on PC1, we speculate that GXXATP10 was a descendant that was formed by the intersection of the East Yungui Plateau population and the western part of the Nanling Mountains population in the past, or a nonnative species that had been introduced from another place.

The Mantel test discovered a significant positive relationship between the genetic and geographical distances in the total distribution (r = 0.47, *p* < 2.2 × 10^−16^), suggesting that isolation by distance plays an important role in genome-wide variation. Combined with the results of *F*_ST_, the phylogenetic tree and the Linkage disequilibrium analysis speculated that the Group I population is closer to the origin center in terms of its genetic distance, which supports that it originated in the Nanling Mountains and the Yungui Plateau before migrating eastward [64].

In the wild, *Dendrobium* species are either epiphytic or lithophytic. Based on this habit, there is no need to compete for the niche of most terrestrial plants. However, people over-excavate and conduct commercial trade for profit, thus causing serious damage to the wild resources [65]. This leads to a decline in the genetic differentiation among the *Dendrobium* species [66].

## 5. Conclusions

In this study, a genetic information database of *D. catenatum* was established, which confirmed that RAD-seq technology has the potential to be applied in the identification of medicinal *Dendrobium* of different origins. The level of genetic diversity in the population of wild *D. catenatum* was relatively low. The 24 wild populations were divided into two groups, and the Group I population is closer to the origin center in terms of its genetic distance. The isolation by distance plays an important role in genome-wide variation. This study provides information for the development of identification technology for medicinal Dendrobium species in order to provide a new perspective for the research of genuine regional drugs. Genetic diversity research provides a theoretical basis for the protection of *Dendrobium*, and it also lays a solid foundation for the breeding of fine *Dendrobium* provenances. Overall, our study provides abundant genomic resources for wild *Dendrobium* species and makes important contributions to its genetic improvement and molecular breeding.

## Figures and Tables

**Table 1:** In this study, the population locations and voucher information of *D. catenatum* and *D. huoshanense*.

Sample Name	Location	Specimen Code	Population Code	Individual Number
*D. catenatum*	Guangze, Fujian Province	Z.J. Liu 10572	FJGZTP01	5
	Liancheng, Fujian Province	Z.J. Liu 10583	FJLCTP02	4
	Pingjiang, Hunan Province	Z.J. Liu 10580	HNPJTP04	5
	Yiyang, Hunan Province	Z.J. Liu 10582	HNYYTP05	5
	Yuanling, Hunan Province	Z.J. Liu 10578	HNYLTP06	5
	Xinning, Hunan Province	Z.J. Liu 9506	HNXNTP36	3
	Lipu, Guangxi Province	Z.J. Liu 10574	GXLPTP07	5
	Gongcheng, Guangxi Province	Z.J. Liu 9497	GXGCTP09	4
	Xing’an, Guangxi Province	Z.J. Liu 9518	GXXATP10	5
	Lishui, Zhengjiang Province	Z.J. Liu 9520	ZJLSTP12	5
	Wuyi, Zhengjiang Province	Z.J. Liu 9511	ZJWYTP32	5
	Quzhou, Zhengjiang Province	Z.J. Liu 9510	ZJQZTP13	5
	Yanshan, Jiangxi Province	Z.J. Liu 10576	JXQSTP14	5
	Xiushui, Jiangxi Province	Z.J. Liu 10573	JXXSTP15	4
	Huichang, Jiangxi Province	Z.J. Liu 10581	JXHCTP16	5
	Xingguo, Jiangxi Province	Z.J. Liu 9517	JXXGTP17	4
	Nanxiong, Guangdong Province	Z.J. Liu 10584	GDNXTP19	5
	Heyuan, Guangdong Province	Z.J. Liu 10585	GDHYTP20	4
	Shaoguan, Guangdong Province	Z.J. Liu 9504	GDSGTP22	5
	Qujing, Yunnan Province	Z.J. Liu 10579	YNQJTP24	4
	Wangzishan, Yunnan Province	Z.J. Liu 7462	WZSTP34	4
	Xianning, Hubei Province	Z.J. Liu 9514	HBXNTP25	5
	Sandu, Guizhou Province	Z.J. Liu 11147	GZSDTP29	4
	Dushan, Guizhou Province	Z.J. Liu 11148	GZDSTP30	4
*D. huoshanense*	Huangshan, Anhui Province	Z.J. Liu 9508	AHHSHS26	5
	Longhushan, Jiangxi Province	Z.J. Liu 9500	JXLHSHS28	5

**Table 2:** The statistical values of genetic diversity within the populations from variants and all of the positions data. (A_P_, private allele number; *H*_O_, observed heterozygosity; *H*_E_, expected heterozygosity; π, nucleotide diversity; *F*_IS_, inbreeding coefficient of an individual relative to the subpopulation.)

Taxon	Population	A_P_	*H*_O_	*H*_E_	π	*F*_IS_	Tajima’s D
*D. catenatum*			0.0992	0.1575	0.1584	0.3836	
	FJGZTP01	3254	0.0786	0.0969	0.1165	0.0725	0.6534
	FJLCTP02	1934	0.0729	0.0833	0.099	0.0496	0.3964
	HNPJTP04	2871	0.0895	0.1131	0.1297	0.0824	0.4221
	HNYYTP05	4263	0.0818	0.1082	0.1223	0.084	0.4411
	HNYLTP06	4689	0.0976	0.1196	0.1371	0.081	0.5556
	GXLPTP07	5512	0.0952	0.1312	0.1484	0.1135	0.4684
	GXGCTP09	2679	0.0965	0.1041	0.1355	0.066	0.7907
	GXXATP10	2940	0.0724	0.0891	0.1074	0.0628	0.8556
	ZJLSTP12	2355	0.0994	0.1182	0.1341	0.0737	0.3298
	ZJQZTP13	3084	0.1123	0.1105	0.1245	0.0256	0.3962
	JXQSTP14	3844	0.123	0.1115	0.1243	0.0053	0.3384
	JXXSTP15	2552	0.0783	0.107	0.1257	0.0922	0.3767
	JXHCTP16	3417	0.0778	0.1037	0.1188	0.0866	0.3404
	JXXGTP17	4215	0.0919	0.096	0.1134	0.039	0.5287
	GDNXTP19	3515	0.0848	0.1022	0.1154	0.0659	0.3163
	GDHYTP20	5221	0.1019	0.1155	0.133	0.0638	0.2443
	GDSGTP22	3845	0.1078	0.0862	0.0961	−0.0208	0.4748
	YNQJTP24	3933	0.1144	0.1153	0.1343	0.039	0.4327
	HBXNTP25	2757	0.0979	0.1057	0.1185	0.0426	0.5005
	GZSDTP29	4461	0.0975	0.1147	0.1326	0.0696	0.4221
	GZDSTP30	9655	0.1516	0.1121	0.1288	−0.0434	0.4646
	ZJWYTP32	2932	0.0885	0.106	0.1191	0.0664	0.2539
	WZSTP34	4820	0.125	0.1165	0.1342	0.0208	0.4762
	HNXNTP36	2967	0.1034	0.0994	0.1218	0.0332	0.4315

## Data Availability

All genomic data generated in this study are deposited in NCBI database (https://www.ncbi.nlm.nih.gov/) (BioProject accession: PRJNA774562). These data will remain private until the related manuscript has been accepted. All other data generated in this manuscript are available from the corresponding author upon reasonable request.

## Supplementary Materials

The following supporting information can be downloaded at: https://www.mdpi.com/article/10.3390/genes13112093/s1, Figure S1: Average clean data size per sample in 24 populations.
