# Supplementary figures and images for: Population Diversity Analysis Provide Insights into Provenance Identification of Dendrobium catenatum
Source: Genes (Basel). 2022 Nov 10;13(11):2093. doi: 10.3390/genes13112093 (PMC9690082; doi:10.3390/genes13112093)

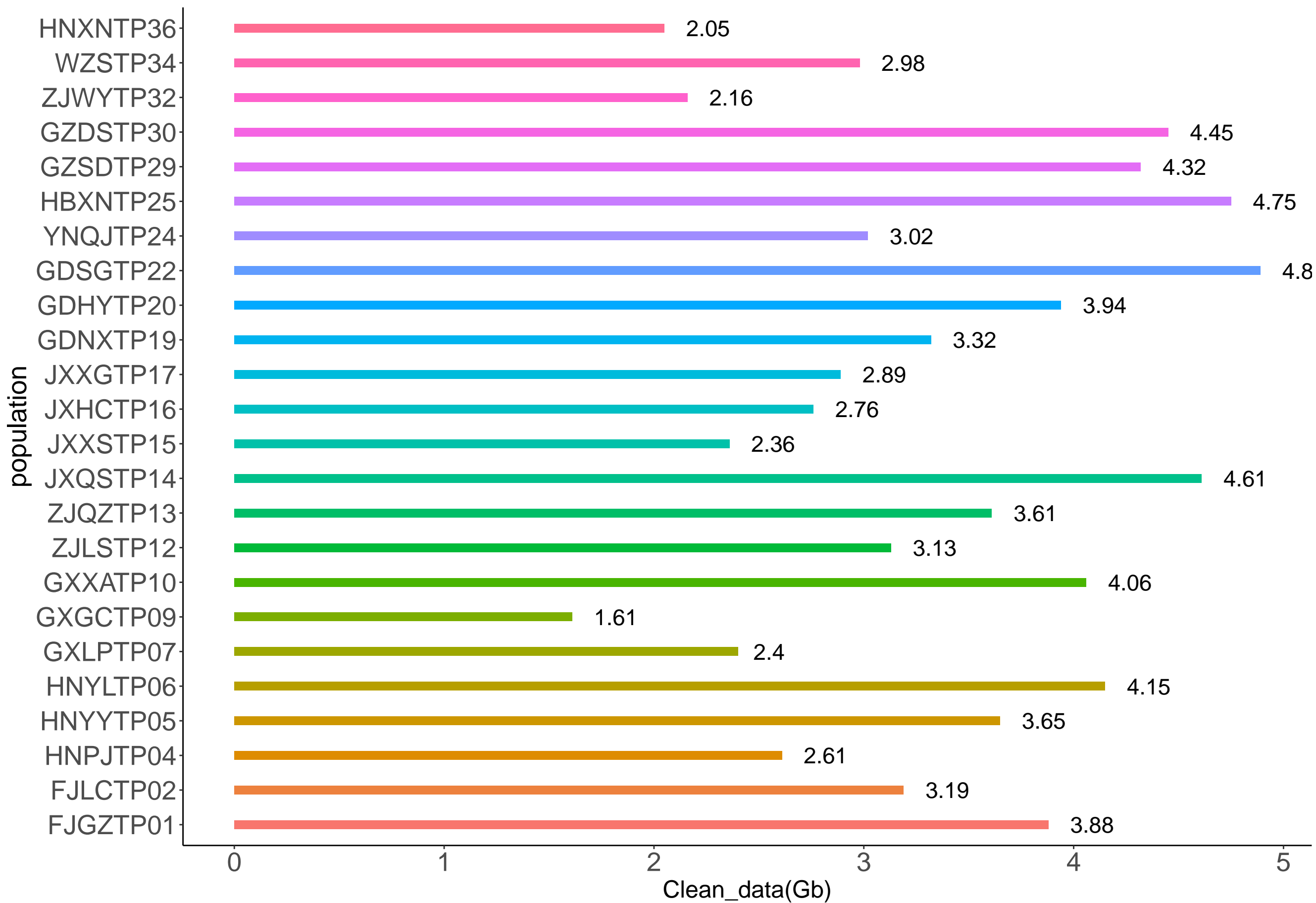

Supplement: Supplementary file 1 [file genes-13-02093-s001.zip › Supplementary Figure S1.pdf]
